# Supplementary material for: Increasing plant diversity with border crops reduces insecticide use and increases crop yield in urban agriculture
Source: eLife. 2018 May 24;7:e35103. doi: 10.7554/eLife.35103 (PMC5967864; doi:10.7554/eLife.35103)
Supplement: Figure 1—source data 1. [file elife-35103-fig1-data1.docx]

**Figure 1—source data 1.** Site, rice pest and predator populations, insecticide use, and yield data for comparison of plant-diversified farms (treatment) and mono-rice farms (control) in Shanghai, China.

| District | Location | Longitude | Latitude | Type of community farms | Survey contents | | | | | | | |
| --- | --- | --- | --- | --- | --- | --- | --- | --- | --- | --- | --- | --- |
|  |  |  |  |  | Pink rice borer trapped in the lamp | Rice brown planthopper trapped in the lamp | Rice leaf roller observed in rice fields | Insecticide use | Grain yield | Predator abundance in the rice fields | Predator abundance on the border crop of soybeans | Predator abundance on the neighboring crops |
| Minhang District | Zhuanqiao | 121.40 | 31.07 | Mono-rice | √ | √ | √ | √ | √ |  |  |  |
|  | Pujiang | 121.51 | 30.99 | Mono-rice | √ | √ | √ | √ | √ | √ |  |  |
| Jiading District | Waigang | 121.16 | 31.35 | Mono-rice | √ | √ | √ | √ | √ | √ |  |  |
|  | Huating | 121.27 | 31.45 | Mono-rice | √ | √ | √ | √ | √ |  |  |  |
| Baoshan District | Luojing | 121.33 | 31.47 | Mono-rice | √ | √ | √ | √ | √ | √ |  |  |
| Pudong District | Chuansha | 121.68 | 31.18 | Mono-rice | √ | √ | √ | √ | √ | √ |  |  |
|  | Shuyuan | 121.86 | 30.94 | Mono-rice | √ | √ | √ | √ | √ |  |  |  |
|  | Xuanqiao | 121.69 | 31.02 | Mono-rice | √ | √ | √ | √ | √ |  |  |  |
|  | Hangtou | 121.74 | 31.06 | Mono-rice | √ | √ | √ | √ | √ | √ |  |  |
|  | Zhuqiao | 121.58 | 31.00 | Mono-rice | √ | √ | √ | √ | √ |  |  |  |
| Fengxian District | Fengcheng | 121.64 | 30.90 | Mono-rice | √ | √ | √ | √ | √ | √ |  |  |
|  | Qingcun | 121.57 | 30.92 | Mono-rice | √ | √ | √ | √ | √ | √ |  |  |
|  | Situan | 121.72 | 30.94 | Mono-rice | √ | √ | √ | √ | √ |  |  |  |
|  | Zhuanhang | 121.37 | 30.90 | Mono-rice | √ | √ | √ | √ | √ | √ |  |  |
| Songjiang District | Maogang | 121.18 | 30.93 | Mono-rice | √ | √ | √ | √ | √ |  |  |  |
|  | Yexie | 121.32 | 30.94 | Mono-rice | √ | √ | √ | √ | √ |  |  |  |
|  | Shihudang | 121.14 | 30.96 | Mono-rice | √ | √ | √ | √ | √ |  |  |  |
|  | Sheshan | 121.13 | 30.96 | Mono-rice | √ | √ | √ | √ | √ | √ |  |  |
| Qingpu District | Jinze | 120.98 | 31.05 | Mono-rice | √ | √ | √ | √ | √ |  |  |  |
|  | Zhujiajiao | 120.97 | 31.06 | Mono-rice | √ | √ | √ | √ | √ |  |  |  |
|  | Liantang | 121.05 | 31.00 | Mono-rice | √ | √ | √ | √ | √ |  |  |  |
|  | Baihe | 121.40 | 31.09 | Mono-rice | √ | √ | √ | √ | √ | √ |  |  |
| Jinshan District | Caojing | 121.40 | 30.79 | Mono-rice | √ | √ | √ | √ | √ |  |  |  |
|  | Tinglin | 121.31 | 30.88 | Mono-rice | √ | √ | √ | √ | √ |  |  |  |
|  | Zhangyan | 121.27 | 30.79 | Mono-rice | √ | √ | √ | √ | √ |  |  |  |
|  | Langxia | 121.18 | 30.77 | Mono-rice | √ | √ | √ | √ | √ |  |  |  |
|  | Zhujing | 121.18 | 30.88 | Mono-rice | √ | √ | √ | √ | √ |  |  |  |
|  | Fengjing | 121.01 | 30.89 | Mono-rice | √ | √ | √ | √ | √ |  |  |  |
| Chongming District | Lvhua | 121.22 | 31.75 | Plant-diversified | √ | √ | √ | √ | √ | √ | √ | √ |
|  | Miaozhen | 121.32 | 31.73 | Plant-diversified | √ | √ | √ | √ | √ | √ | √ | √ |
|  | Baozhen | 121.61 | 31.52 | Plant-diversified | √ | √ | √ | √ | √ | √ | √ | √ |
|  | Zhongxing | 121.77 | 31.5 | Plant-diversified | √ | √ | √ | √ | √ | √ | √ | √ |
|  | Jianshe | 121.46 | 31.62 | Plant-diversified | √ | √ | √ | √ | √ | √ | √ | √ |
|  | Shuxin | 121.56 | 31.57 | Plant-diversified | √ | √ | √ | √ | √ | √ | √ | √ |

Note: Shuyuan, Xuanqiao, Hangtou and Zhuqiao Towns belonged to Nanhui Distrcit which was integrated into Pudong District in 2009
