# Supplementary material for: Increasing plant diversity with border crops reduces insecticide use and increases crop yield in urban agriculture
Source: eLife. 2018 May 24;7:e35103. doi: 10.7554/eLife.35103 (PMC5967864; doi:10.7554/eLife.35103)
Supplement: Figure 1—source data 2. — The crosses indicate, for each insecticide, when they were used. [file elife-35103-fig1-data2.docx]

**Figure 1—source data 2.** Chemical insecticides applied to control the main insect pests on plant-diversified farms and mono-rice farms in Shanghai, China from 2001 to 2015. The crosses indicate, for each insecticide, when they were used.

| Insecticide  Name | Active ingredients of insecticides | 2001 | 2002 | 2003 | 2004 | 2005 | 2006 | 2007 | 2008 | 2009 | 2010 | 2011 | 2012 | 2013 | 2014 | 2015 |
| --- | --- | --- | --- | --- | --- | --- | --- | --- | --- | --- | --- | --- | --- | --- | --- | --- |
| Buprofezin | 2-(tert-Butylimino)-3-isopropyl-5-  phenylperhydro-1, 3, 5-thidiazin-4-one | + | + | + | + | + | + | + | + | + | + | + | + | + | + | + |
| Monosultap | sodiumS-[2-(dimethylamino)-3-  (sulfosulfanyl)propyl] sulfurothioate | + | + | + | + | + | + | + | + | + |  |  |  |  |  |  |
| Imidacloprid | 1-((6-Chloro-3-pyridinyl)methyl)-N-  nitro-2-imidazolidinimine | + | + | + | + | + | + | + |  |  | + |  |  |  |  |  |
| Fipronil | 5-amino-1-[2,6-dichloro-4-(trifluoromethyl)  phenyl]-4-[(trifluoromethyl)thio] | + | + | + | + | + | + | + |  |  |  |  |  |  |  |  |
| Triazophos | O, O-diethyl-O-(1-phenyl-1,2,4-triazol  -3-yl) phosphorothioate | + | + | + | + | + | + |  |  |  |  |  |  |  |  |  |
| Hexaflumuron | 1-(3,5-dichloro-4-(1,1,2,2-tetrafluoroethoxy)  phenyl)-3-(2,6-difluorobenzoyl) | + | + | + | + | + |  |  |  |  |  |  |  |  |  |  |
| Dimethacarb | 3,4,5-Trimethylphenyl  N-methylcarbamate | + | + | + | + | + | + |  |  |  |  |  |  |  |  | + |
| Chlorpyrifos | O,O-Diethyl-O-(3,5,6-trichloro-  2-pyridyl) phosphorothioate |  |  |  |  | + | + | + | + | + | + | + | + | + | + | + |
| Phoxim | Benzoyl cyanide-O-  (diethoxyphosphinothioyl)oxime |  |  |  |  |  | + | + | + | + | + |  |  |  |  |  |
| Profenofos | O, O-diethyl-S-propyl  dithiophosphate |  |  |  |  |  |  | + |  |  |  |  |  |  |  |  |
| Pymetrozine | 6-Methyl-4-((pyridin-3-ylMethylene)aMino)  -4,5-dihydro-1,2,4-triazin-3(2H)-one |  |  |  |  |  |  | + | + | + | + | + | + | + | + | + |
| Emamectin-benzoate | Avermectin B1, 4-deoxy-4-  (methylamino)-, (4R)-, benzoate (salt) |  |  |  |  |  |  |  |  |  | + | + | + | + | + |  |
| Ethofenprox | 1-((2-(4-ethoxyphenyl)-2-methylpropoxy)  methyl)-3-phenoxybenzene |  |  |  |  |  |  |  |  |  | + |  |  |  |  |  |
| Nitenpyram | N-ethyl-N'-methyl-2-nitroethene-1,  1-diamine |  |  |  |  |  |  |  |  |  |  |  |  | + | + |  |
| Flutolanil | alpha,alpha,alpha-trifluoro-  3’-isopropoxy-o-toluanilide |  |  |  |  |  |  |  |  |  |  |  |  |  |  | + |
| Indoxacarb | methyl 7-chloro-2-[methoxycarbonyl-[4-  (trifluoromethoxy) phenyl]carbamoyl]  -3,5-dihydroindeno[1,2-e][1,3,4]  oxadiazine-4a-carboxylate |  |  |  |  |  |  |  |  |  |  |  |  |  |  | + |
