# Supplementary material for: Increasing plant diversity with border crops reduces insecticide use and increases crop yield in urban agriculture
Source: eLife. 2018 May 24;7:e35103. doi: 10.7554/eLife.35103 (PMC5967864; doi:10.7554/eLife.35103)
Supplement: Figure 2—source data 1. [file elife-35103-fig2-data1.docx]

## Figure 2—source data 1. Pink rice borer: mean and standard deviation (individual per lamp per year) from the 15-year monitoring data, stratified by year and farm type.

| Year | Mono-rice  mean (s.d.) | Plant-diversified  mean (s.d.) |
| --- | --- | --- |
| 2001 | 1.18 (0.71) | 0.93 (0.21) |
| 2002 | 1.55 (0.75) | 1.03 (0.31) |
| 2003 | 1.74 (1.08) | 1.43 (0.46) |
| 2004 | 4.25 (1.50) | 3.34 (1.37) |
| 2005 | 4.30 (1.12) | 2.28 (0.69) |
| 2006 | 4.05 (0.65) | 2.61 (0.64) |
| 2007 | 3.01 (1.17) | 2.47 (0.77) |
| 2008 | 3.86 (0.65) | 2.24 (0.52) |
| 2009 | 2.67 (0.55) | 2.23 (0.37) |
| 2010 | 4.01 (0.62) | 2.96 (0.52) |
| 2011 | 5.10 (2.08) | 2.87 (0.69) |
| 2012 | 2.92 (0.98) | 2.33 (0.69) |
| 2013 | 2.35 (0.56) | 1.65 (0.45) |
| 2014 | 3.84 (1.63) | 2.66 (0.43) |
| 2015 | 3.22 (0.54) | 2.30 (0.43) |
