# Supplementary material for: Increasing plant diversity with border crops reduces insecticide use and increases crop yield in urban agriculture
Source: eLife. 2018 May 24;7:e35103. doi: 10.7554/eLife.35103 (PMC5967864; doi:10.7554/eLife.35103)
Supplement: Figure 2—source data 2. [file elife-35103-fig2-data2.docx]

### Figure 2—source data 2. Rice brown planthopper: mean and standard deviation (individual per lamp per year) from the 15-year monitoring data, stratified by year and farm type.

| Year | Mono-rice  mean (s.d.) | Plant-diversified  mean (s.d.) |
| --- | --- | --- |
| 2001 | 3.18 (1.57) | 2.39 (1.30) |
| 2002 | 9.69 (2.37) | 7.42 (1.82) |
| 2003 | 10.31 (2.70) | 7.18 (1.70) |
| 2004 | 1.46 (0.81) | 1.03 (0.62) |
| 2005 | 26.20 (12.82) | 19.21 (8.25) |
| 2006 | 345.81 (193.16) | 240.16 (40.13) |
| 2007 | 51.40 (17.95) | 42.78 (25.93) |
| 2008 | 4.05 (2.18) | 2.87 (1.05) |
| 2009 | 2.37 (1.99) | 1.74 (0.60) |
| 2010 | 13.47 (9.56) | 10.49 (2.08) |
| 2011 | 1.87 (1.61) | 0.27 (0.11) |
| 2012 | 5.78 (3.57) | 3.14 (1.83) |
| 2013 | 2.78 (1.36) | 2.04 (1.48) |
| 2014 | 4.65 (1.49) | 2.32 (1.44) |
| 2015 | 1.52 (1.53) | 1.26 (0.47) |
