# Supplementary material for: Increasing plant diversity with border crops reduces insecticide use and increases crop yield in urban agriculture
Source: eLife. 2018 May 24;7:e35103. doi: 10.7554/eLife.35103 (PMC5967864; doi:10.7554/eLife.35103)
Supplement: Figure 2—source data 3. [file elife-35103-fig2-data3.docx]

## Figure 2—source data 3. Rice leaf roller: mean and standard deviation (individual per ha per year) from the 15-year monitoring data, stratified by year and farm type.

| Year | Mono-rice  mean (s.d.) | Plant-diversified  mean (s.d.) |
| --- | --- | --- |
| 2001 | 2,510.61 (878.46) | 1,851.15 (229.97) |
| 2002 | 5,464.89 (2,271.05) | 4,153.06 (700.32) |
| 2003 | 7,172.96 (1,882.21) | 6,340.96 (1,172.19) |
| 2004 | 6,657.24 (1,608.72) | 5,198.26 (906.03) |
| 2005 | 13,380.00 (2,990.70) | 10,164.19 (1,299.94) |
| 2006 | 8,117.65 (1,578.23) | 6,042.35 (1,592.22) |
| 2007 | 21,030.20 (4,609.91) | 16,400.69 (6,725.08) |
| 2008 | 9,643.97 (3,055.90) | 7,362.77 (1,302.30) |
| 2009 | 6,174.04 (1,106.50) | 5,616.81 (752.29) |
| 2010 | 5,241.18 (1,507.18) | 4,637.48 (1,105.61) |
| 2011 | 4,396.18 (892.60) | 3,755.91 (437.37) |
| 2012 | 2,816.71 (996.04) | 2,326.03 (815.45) |
| 2013 | 5,626.69 (960.55) | 4,694.80 (888.30) |
| 2014 | 2,117.81 (525.74) | 1,723.50 (326.62) |
| 2015 | 2,815.06 (589.62) | 1,862.50 (482.58) |

## 
