# Supplementary material for: Increasing plant diversity with border crops reduces insecticide use and increases crop yield in urban agriculture
Source: eLife. 2018 May 24;7:e35103. doi: 10.7554/eLife.35103 (PMC5967864; doi:10.7554/eLife.35103)
Supplement: Figure 2—source data 4. — ‘/' denotes that there is no Economic Injury Level for rice leaf roller in grain-filling stage of rice. [file elife-35103-fig2-data4.docx]

**Figure 2—source data 4.** Economic Injury Levels of pink rice borers, rice brown planthoppers and rice leaf rollers issued by Shanghai Agricultural Technology Extension and Service Center (SATESC). “/” denotes that there is no Economic Injury Level for rice leaf roller in grain-filling stage of rice.

| Rice growth stage | Pink rice borer  (damaged cluster per 100 sampling clusters) | Rice brown planthopper  (10 thousand individuals per ha) | Rice leaf roller  (percentage of damaged plants per 100 sampling plants) |
| --- | --- | --- | --- |
| Tillering stage | 5 | 75 | 20% |
| Elongation stage | 5 | 150 | 15% |
| Booting stage | 5 | 225 | 10% |
| Grain-filling stage | 5 | 300 | / |
