# Supplementary material for: Increasing plant diversity with border crops reduces insecticide use and increases crop yield in urban agriculture
Source: eLife. 2018 May 24;7:e35103. doi: 10.7554/eLife.35103 (PMC5967864; doi:10.7554/eLife.35103)
Supplement: Figure 3—source data 1. [file elife-35103-fig3-data1.docx]

## Figure 3—source data 1. Predator abundances: mean and standard deviation (individual per 100 rice clusters per year) from the 15-year monitoring data, stratified by year and farm type.

| Year | Mono-rice  mean (s.d.) | Plant-diversified  mean (s.d.) |
| --- | --- | --- |
| 2007 | 81.87 (5.48) | 90.15 (2.61) |
| 2008 | 82.78 (3.83) | 93.48 (2.56) |
| 2009 | 82.94 (3.90) | 94.75 (8.19) |
| 2010 | 82.85 (4.48) | 95.60 (6.94) |
| 2011 | 84.92 (3.46) | 99.20 (4.23) |
| 2012 | 85.71 (1.87) | 100.88 (3.45) |
| 2013 | 84.06 (4.63) | 98.48 (6.32) |
| 2014 | 86.78 (4.85) | 102.16 (5.32) |
| 2015 | 87.81 (5.43) | 101.78 (9.09) |

## 
