# Supplementary material for: Increasing plant diversity with border crops reduces insecticide use and increases crop yield in urban agriculture
Source: eLife. 2018 May 24;7:e35103. doi: 10.7554/eLife.35103 (PMC5967864; doi:10.7554/eLife.35103)
Supplement: Figure 5—source data 1. [file elife-35103-fig5-data1.docx]

## Figure 5—source data 1. The amount of commercial insecticide and active ingredient sprayed per crop: mean and standard deviation (kg•ha^-1^) from the 15-year monitoring data, stratified by year and farm type.

| Year | Mono-rice  mean (s.d.) | Plant-diversified  mean (s.d.) | Mono-rice  mean (s.d.) | Plant-diversified  mean (s.d.) |
| --- | --- | --- | --- | --- |
|  | Commercial | Commercial | Active ingredient | Active ingredient |
| 2001 | 9.45 (0.44) | 9.30 (0.47) | 3.54 (0.11) | 3.50 (0.14) |
| 2002 | 8.78 (0.42) | 8.40 (0.37) | 3.46 (0.12) | 3.44 (0.11) |
| 2003 | 10.76 (0.58) | 9.68 (0.37) | 3.72 (0.17) | 2.72 (0.11) |
| 2004 | 10.01 (0.35) | 8.70 (0.47) | 3.77 (0.07) | 3.58 (0.09) |
| 2005 | 14.49 (0.48) | 11.90 (0.31) | 4.49 (0.10) | 3.99 (0.06) |
| 2006 | 13.16 (0.58) | 10.24 (0.09) | 4.63 (0.12) | 4.02 (0.01) |
| 2007 | 10.76 (0.19) | 8.47 (0.19) | 3.86 (0.05) | 2.95 (0.05) |
| 2008 | 9.19 (0.19) | 7.42 (0.19) | 3.57 (0.05) | 2.90 (0.05) |
| 2009 | 6.00 (0.14) | 4.47 (0.12) | 2.32 (0.03) | 1.71 (0.03) |
| 2010 | 8.11 (0.58) | 6.42 (0.44) | 1.42 (0.06) | 1.16 (0.04) |
| 2011 | 6.28 (0.16) | 4.95 (0.18) | 1.04 (0.06) | 0.78 (0.07) |
| 2012 | 7.95 (0.16) | 6.42 (0.12) | 1.94 (0.04) | 1.41 (0.03) |
| 2013 | 9.14 (0.47) | 7.46 (0.37) | 3.02 (0.14) | 1.95 (0.11) |
| 2014 | 7.33 (0.21) | 6.06 (0.18) | 1.29 (0.12) | 0.57 (0.07) |
| 2015 | 7.27 (0.10) | 6.19 (0.09) | 2.04 (0.05) | 0.69 (0.05) |
