# Supplementary material for: Increasing plant diversity with border crops reduces insecticide use and increases crop yield in urban agriculture
Source: eLife. 2018 May 24;7:e35103. doi: 10.7554/eLife.35103 (PMC5967864; doi:10.7554/eLife.35103)
Supplement: Figure 5—source data 2. [file elife-35103-fig5-data2.docx]

## Figure 5—source data 2. The number of insecticide spray per crop: mean and standard deviation from the 15-year monitoring data, stratified by year and farm type.

| Year | Mono-rice  mean (s.d.) | Plant-diversified  mean (s.d.) |
| --- | --- | --- |
| 2001 | 6.00 (0.53) | 6.00 (0.63) |
| 2002 | 5.25 (0.46) | 5.17 (0.41) |
| 2003 | 8.12 (0.64) | 7.17 (0.41) |
| 2004 | 5.75 (0.46) | 5.00 (0.63) |
| 2005 | 8.12 (0.64) | 6.17 (0.41) |
| 2006 | 9.12 (0.64) | 7.17 (0.41) |
| 2007 | 9.12 (0.64) | 8.00 (0.63) |
| 2008 | 8.88 (0.64) | 8.00 (0.63) |
| 2009 | 7.25 (0.46) | 6.17 (0.41) |
| 2010 | 8.12 (0.64) | 6.00 (0.63) |
| 2011 | 5.12 (0.35) | 4.17 (0.41) |
| 2012 | 6.00 (0.53) | 5.17 (0.41) |
| 2013 | 6.38 (0.52) | 5.17 (0.41) |
| 2014 | 5.75 (0.46) | 4.83 (0.41) |
| 2015 | 6.25 (0.46) | 5.17 (0.41) |
