# Supplementary material for: Increasing plant diversity with border crops reduces insecticide use and increases crop yield in urban agriculture
Source: eLife. 2018 May 24;7:e35103. doi: 10.7554/eLife.35103 (PMC5967864; doi:10.7554/eLife.35103)
Supplement: Figure 5—source data 3. — All data are mean values from 2001 to 2015; average grain price is 2.163 RMB per kilogram, and average soybean price is 3.873 RMB per kilogram; about 3% of one-hectare rice field was taken by soybeans in the field ridge, and soybean yield is about 25.0 kilograms per hectare rice field. Means ± SE. [file elife-35103-fig5-data3.docx]

**Figure 5—source data 3.** Cost–benefit analysis of plant-diversified farms compared with mono-rice farms. All data are mean values from 2001 to 2015; average grain price is 2.163 RMB per kilogram, and average soybean price is 3.873 RMB per kilogram; about 3% of one-hectare rice field was taken by soybeans in the field ridge, and soybean yield is about 25.0 kilograms per hectare rice field. Means±SE.

| Item | Index | Plant-diversified farms | | Mono-rice farms |  |
| --- | --- | --- | --- | --- | --- |
| Cost | | Cost of insecticide entities sprayed in fields (RMB • ha^-1^ • year^-1^) | | 569.02±45.61 | 681.09±59.08 |
|  | | Cost of labor force of insecticide sprays in fields (RMB • ha^-1^ • year^-1^) | | 535.83±47.49 | 640.66±60.61 |
|  | | Cost of soybean seed entities planted on paddy field ridge (RMB • ha^-1^ • year^-1^) | | 5.81±0.52 | 0 |
|  | | Cost of planting and harvesting soybeans on paddy field ridge (RMB • ha^-1^ • year^-1^) | | 11.07±1.08 | 0 |
| Benefit | | Value of grain yield (RMB • ha^-1^ • year^-1^) | | 18138.34±1701.01 | 17859.44±1659.31 |
|  | | Value of harvested soybeans on paddy field ridge (RMB • ha^-1^ • year^-1^) | | 96.83±8.74 | 0 |
| Net benefit | | Benefit – cost | | 17113.44±1645.50 | 16537.69±1572.06 |
| Relative advantage | | Net benefit of treatment–net benefit of control (RMB • ha^-1^ • year^-1^) | | 575.75±116.00 | / |
| Percent relative advantage | | (Net benefit of treatment–net benefit of control) / (net benefit of control) × 100 (%) | | 3.48±0.79 | / |
