# Supplementary material for: Increasing plant diversity with border crops reduces insecticide use and increases crop yield in urban agriculture
Source: eLife. 2018 May 24;7:e35103. doi: 10.7554/eLife.35103 (PMC5967864; doi:10.7554/eLife.35103)
Supplement: Figure 5—source data 4. [file elife-35103-fig5-data4.docx]

## Figure 5—source data 4. Grain yield: mean and standard deviation (kg•ha^-1^) from the 15-year monitoring data, stratified by year and farm type.

| Year | Mono-rice  mean (s.d.) | Plant-diversified  mean (s.d.) |
| --- | --- | --- |
| 2001 | 7,948.38 (133.59) | 7,692.17 (202.73) |
| 2002 | 7,736.25 (217.62) | 7,530.17 (310.43) |
| 2003 | 7,829.75 (418.81) | 7,766.33 (311.34) |
| 2004 | 8,073.00 (223.27) | 8,307.50 (325.94) |
| 2005 | 7,882.62 (409.72) | 8,273.00 (233.87) |
| 2006 | 8,159.25 (75.16) | 8,296.17 (156.10) |
| 2007 | 8,093.62 (350.55) | 8,451.00 (243.72) |
| 2008 | 8,230.38 (188.82) | 8,236.17 (227.73) |
| 2009 | 8,274.75 (402.26) | 8,599.00 (175.96) |
| 2010 | 8,344.25 (329.19) | 8,217.00 (235.52) |
| 2011 | 8,319.12 (124.41) | 8,569.17 (177.86) |
| 2012 | 8,485.88 (115.79) | 8,562.00 (192.77) |
| 2013 | 8,441.12 (115.93) | 8,665.33 (201.23) |
| 2014 | 8,432.12 (308.07) | 8,554.17 (182.73) |
| 2015 | 8,519.88 (213.39) | 8,655.00 (240.78) |
