# Supplementary material for: Increasing plant diversity with border crops reduces insecticide use and increases crop yield in urban agriculture
Source: eLife. 2018 May 24;7:e35103. doi: 10.7554/eLife.35103 (PMC5967864; doi:10.7554/eLife.35103)
Supplement: Figure 6—source data 1. [file elife-35103-fig6-data1.docx]

## Figure 6—source data 1. Stem borer: mean and standard deviation (individual per 100 rice clusters) from the common-location-experiments, stratified by year, farm identity, and farm type.

| Year | Farm identity | Mono-rice  mean (s.d.) | Plant-diversified  mean (s.d.) |
| --- | --- | --- | --- |
| 2009 | 1 | 3.37 (0.40) | 2.73 (0.32) |
| 2010 | 1 | 3.57 (0.42) | 3.07 (0.32) |
| 2013 | 2 | 4.30 (0.00) | 3.57 (0.12) |
| 2014 | 2 | 3.77 (0.38) | 3.30 (0.46) |
