# Supplementary material for: Increasing plant diversity with border crops reduces insecticide use and increases crop yield in urban agriculture
Source: eLife. 2018 May 24;7:e35103. doi: 10.7554/eLife.35103 (PMC5967864; doi:10.7554/eLife.35103)
Supplement: Figure 6—source data 2. [file elife-35103-fig6-data2.docx]

## Figure 6—source data 2. Rice plant-hopper: mean and standard deviation (individual per 100 rice clusters) from the common-location-experiments, stratified by year, farm identity, and farm type.

| Year | Farm identity | Mono-rice  mean (s.d.) | Plant-diversified  mean (s.d.) |
| --- | --- | --- | --- |
| 2009 | 1 | 32.73 (1.76) | 27.13 (1.30) |
| 2010 | 1 | 40.50 (3.41) | 34.33 (2.56) |
| 2013 | 2 | 25.47 (1.50) | 20.67 (2.31) |
| 2014 | 2 | 33.20 (1.92) | 28.00 (1.47) |
