# Supplementary material for: Increasing plant diversity with border crops reduces insecticide use and increases crop yield in urban agriculture
Source: eLife. 2018 May 24;7:e35103. doi: 10.7554/eLife.35103 (PMC5967864; doi:10.7554/eLife.35103)
Supplement: Figure 6—source data 4. [file elife-35103-fig6-data4.docx]

## Figure 6—source data 4. Predator: mean and standard deviation (individual per 100 rice clusters) from the common-location-experiments, stratified by year, farm identity, and farm type.

| Year | Farm identity | Mono-rice  mean (s.d.) | | Plant-diversified  mean (s.d.) |
| --- | --- | --- | --- | --- |
| 2009 | 1 | | 80.23 (0.91) | 91.23 (3.58) |
| 2010 | 1 | | 82.37 (3.74) | 94.57 (2.95) |
| 2013 | 2 | | 81.53 (1.17) | 88.73 (1.78) |
| 2014 | 2 | | 80.13 (2.64) | 90.80 (4.75) |
