# Supplementary material for: Increasing plant diversity with border crops reduces insecticide use and increases crop yield in urban agriculture
Source: eLife. 2018 May 24;7:e35103. doi: 10.7554/eLife.35103 (PMC5967864; doi:10.7554/eLife.35103)
Supplement: Figure 6—source data 5. [file elife-35103-fig6-data5.docx]

## Figure 6—source data 5. Insecticide amount: mean and standard deviation (kg•ha^-1^) from the common-location-experiments, stratified by year, farm identity, and farm type.

| Year | Farm identity | Mono-rice  mean (s.d.) | Plant-diversified  mean (s.d.) |
| --- | --- | --- | --- |
| 2009 | 1 | 6.47 (0.35) | 5.25 (0.35) |
| 2010 | 1 | 7.07 (0.35) | 6.41 (0.36) |
| 2013 | 2 | 8.21 (0.26) | 7.16 (0.26) |
| 2014 | 2 | 8.12 (0.39) | 6.83 (0.69) |
