# Supplementary material for: Increasing plant diversity with border crops reduces insecticide use and increases crop yield in urban agriculture
Source: eLife. 2018 May 24;7:e35103. doi: 10.7554/eLife.35103 (PMC5967864; doi:10.7554/eLife.35103)
Supplement: Figure 6—source data 6. [file elife-35103-fig6-data6.docx]

## Figure 6—source data 6. Yield: mean and standard deviation (kg•ha^-1^) from the common-location-experiments, stratified by year, farm identity, and farm type.

| Year | Farm identity | Mono-rice  mean (s.d.) | Plant-diversified  mean (s.d.) |
| --- | --- | --- | --- |
| 2009 | 1 | 8,321.23 (100.89) | 8,478.27 (126.07) |
| 2010 | 1 | 8,365.73 (97.32) | 8,568.60 (95.17) |
| 2013 | 2 | 8,538.30 (106.55) | 8,727.20 (94.19) |
| 2014 | 2 | 8,452.67 (176.77) | 8,666.60 (84.44) |
