# Supplementary material for: Increasing plant diversity with border crops reduces insecticide use and increases crop yield in urban agriculture
Source: eLife. 2018 May 24;7:e35103. doi: 10.7554/eLife.35103 (PMC5967864; doi:10.7554/eLife.35103)
Supplement: Transparent reporting form [file elife-35103-transrepform.docx]

***eLife’s* transparent reporting form**

We encourage authors to provide detailed information *within their submission* to facilitate the interpretation and replication of experiments. Authors can upload supporting documentation to indicate the use of appropriate reporting guidelines for health-related research (see [EQUATOR Network](http://www.equator-network.org/%20)), life science research (see the [BioSharing Information Resource](https://biosharing.org/)), or the [ARRIVE guidelines](http://www.plosbiology.org/article/info:doi/10.1371/journal.pbio.1000412) for reporting work involving animal research. Where applicable, authors should refer to any relevant reporting standards documents in this form.

If you have any questions, please consult our Journal Policies and/or contact us: [editorial@elifesciences.org](mailto:editorial@elifesciences.org).

**Sample-size estimation**

- You should state whether an appropriate sample size was computed when the study was being designed
- You should state the statistical method of sample size computation and any required assumptions
- If no explicit power analysis was used, you should describe how you decided what sample (replicate) size (number) to use

Please outline where this information can be found within the submission (e.g., sections or figure legends), or explain why this information doesn’t apply to your submission:

We selected 28 community farms on which was the sole crop as the control (120°54’–121°51’E, 30°47’–31°28’N), which is the standard practice. We also set up 6 plant-diversified community farms in the suburb of Chongming Island as the treatment (121°12’–121°46’E, 31°32’–31°49’N). We monitored these 34 community farms in mega-urban Shanghai, China, from 2001 to 2015. This information can be found within the submission in Figure 1, Figure 1—source data 1 and Figure 1—source data 2.

Because the mono-rice and diversified farms in the 15-year study were located in different suburban areas of Shanghai, we also performed two, more highly controlled, 2-year experiments in which both treatments were performed at the same locations. One experiment was conducted in Xinchang Town, Pudong district (121°38’E, 31°01’N), in 2009 and 2010 and the other in Sanxing Town, Chongming district, (121°17’E, 31°44’N) in 2013 and 2014. For each site, the experiment was a randomized block design with three replicates.

**Replicates**

- You should report how often each experiment was performed
- You should include a definition of biological versus technical replication
- The data obtained should be provided and sufficient information should be provided to indicate the number of independent biological and/or technical replicates
- If you encountered any outliers, you should describe how these were handled
- Criteria for exclusion/inclusion of data should be clearly stated
- High-throughput sequence data should be uploaded before submission, with a private link for reviewers provided (these are available from both GEO and ArrayExpress)

Please outline where this information can be found within the submission (e.g., sections or figure legends), or explain why this information doesn’t apply to your submission:

Our monitoring experiment was performed from 2001 to 2015, in each year there are 28 community farms (belonging to 8 districts of Shanghai, China) (i.e., 28 replicates for mono-rice farms), and 6 community farms (belonging to one district-Chongming District, Shanghai , China) (i.e., 6 replicates for plant-diversified farms). This information can be found within the submission in Figure 1, Figure 1—source data 1 and Figure 1—source data 2.

Our common location experiments were performed in 2009 and 2010 and in 2013 and 2014 respectively. For each site, the experiment was a randomized block design with three replicates. Each block contained two treatments (two plots)—one plot for plant-diversified farming and the other for mono-rice farming (50-55 m × 30-35 m plot size in Xinchang, and 55-60 m × 35-40 m in Sanxing).

**Statistical reporting**

- Statistical analysis methods should be described and justified
- Raw data should be presented in figures whenever informative to do so (typically when N per group is less than 10)
- For each experiment, you should identify the statistical tests used, exact values of N, definitions of center, methods of multiple test correction, and dispersion and precision measures (e.g., mean, median, SD, SEM, confidence intervals; and, for the major substantive results, a measure of effect size (e.g., Pearson's r, Cohen's d)
- Report exact p-values wherever possible alongside the summary statistics and 95% confidence intervals. These should be reported for all key questions and not only when the p-value is less than 0.05.

Please outline where this information can be found within the submission (e.g., sections or figure legends), or explain why this information doesn’t apply to your submission:

The monitoring studies involved 34 farms belonging to 9 districts of Shanghai, and each farm had longitudinal repeated measurements across the 15-year study period. Due to the longitudinal nature of the data, we used mixed-effects models, a widely adopted method for analyzing data with repeated measurements (Bolker et. al., 2009), to analyse the effects of the community farm types (plant-diversified and mono-rice farms) on the following 8 response variables: numbers of pink rice borers and rice brown plant-hoppers trapped in the lamp, the population density of rice leaf roller observed in rice fields, the abundance of predators observed in the rice fields, the number of insecticide sprays, the amount of commercial and active ingredient insecticide sprayed, and grain yield. For predator abundance in the rice fields, the data used for analysis involved measurements from 6-10 mono-rice farms and 3-6 diversified-farms collected from 2007 to 2015. For the other 7 outcome variables from 2001 to 2015, measurements for plant-diversified farming practices were obtained from the 6 community farms within Chongming district, and measurements from the 28 mono-rice farms were averaged at the 8 district levels except for Chongming district (resulting in eight averaged measurements for mono-rice farming and six measurements for plant-diversified farming, collected annually across the 15-year monitoring period).

To account for the correlation among the outcomes at neighboring years within each farm identity, the mixed-effect model incorporates random intercepts and random coefficients for the polynomial year terms to model variation in yearly trends among the farms. Third-degree polynomials were used to model the yearly trends within each farm as the yearly trends were nonlinear for the majority of the responses (Figures 2, 3, 5). Altogether, year is treated as a categorical variable in the fixed-effect component of the model for adjustment of the overall year effect across all locations, and as a continuous variable in the random component for adjusting the within-farm yearly trends. To determine the significance of farm type, linear mixed effect models with fixed-effects farm type, year, and interaction between farm type and year were first used to test whether the interaction effect was significant using model comparison tests. If the interaction effect was not significant, the interaction term was dropped from the model, and the significance of farm type determined using a model with only farm type and year as fixed-effects. If the interaction term was significant, the farm type effect was analyzed in conjunction with the interaction effect. The numbers of the three pest groups were log10(X + 1) transformed to reduce the influence of a few outlying observations.

In the common-location experiments, replicated measurements were obtained from two farms, and each farm had measurements collected over two years. Here we also used a mixed-effects model to analyse the effects of the rice field types (plant-diversified fields and mono-rice fields) on the densities of rice plant-hoppers, stem borers, leaf rollers and predators sampled in rice field plots, the amount of insecticide sprays, and grain yield. In this mixed-effect model, the random-effects component contains varying intercepts, farm type effects, and year effects for the farm identities. The significance of the farm type effect was determined by model comparison tests, where the full model with the farm type factor was compared with the reduced model with the farm type factor removed.

Parametric bootstrap tests with 2500 bootstrap samples were used for significance testing (Halekoh et al. 2014). The standard significance level 0.05 and a marginal significance level 0.10 were considered. R version 3.3.0 was used for the above analyses.

An economic cost–benefit analysis was conducted according to Gurr et al. (2016), in which we considered the insecticide material costs in each treatment, the cost of seeds used to establish border and neighboring crops in the plant-diversified farming treatment. The analysis also reflected a labor cost for spraying insecticides, planting and soybean harvesting. The benefit was focused on the value of grain yield and the soybeans harvested on paddy field ridges. Other material inputs and labor costs were not included, as these were not consistent across treatments. The grain and soybean price per kilogram and the labor force was based on the average price in Shanghai each year. The economic costs of biodiversity loss, environmental pollution and human health caused by the negative effects of pesticides could not be included.

For the common-location experiment data analysis, simulation-based power analyses were performed for marginally significant responses to farm type at the standard significance level of 0.05 for the abundances of rice plant-hoppers, leaf rollers and predators and for grain yield. Using the estimated effect size from the fitted mixed-effects model, the statistical power was estimated for number of experimental sites from 2 to 8, number of years from 2 to 8, and number of replicates within each farm type-site-year combination from 3 to 38. Statistical power was estimated based on 200 simulations. The target statistical power was 80%, and the R package “SIMR” (Green et al. 2016) was used for power analyses. Due to the costs and manpower required, additional replication of the common-location experiments was not possible.

(For large datasets, or papers with a very large number of statistical tests, you may upload a single table file with tests, Ns, etc., with reference to sections in the manuscript.)

**Group allocation**

- Indicate how samples were allocated into experimental groups (in the case of clinical studies, please specify allocation to treatment method); if randomization was used, please also state if restricted randomization was applied
- Indicate if masking was used during group allocation, data collection and/or data analysis

Please outline where this information can be found within the submission (e.g., sections or figure legends), or explain why this information doesn’t apply to your submission:

There is no clinical studies or group allocation.

**Additional data files (“source data”)**

- We encourage you to upload relevant additional data files, such as numerical data that are represented as a graph in a figure, or as a summary table
- Where provided, these should be in the most useful format, and they can be uploaded as “Source data” files linked to a main figure or table
- Include model definition files including the full list of parameters used
- Include code used for data analysis (e.g., R, MatLab)
- Avoid stating that data files are “available upon request”

Please indicate the figures or tables for which source data files have been provided:

Figure 1—source data 1. Site, rice pest and predator populations, insecticide use, and yield data for comparison of plant-diversified farms (treatment) and mono-rice farms (control) in Shanghai, China.

Figure 1—source data 2. Chemical insecticides applied to control the main insect pests on plant-diversified farms and mono-rice farms in Shanghai, China from 2001 to 2015. The crosses indicate, for each insecticide, when they were used.

Figure 2—source data 1. Pink rice borer: mean and standard deviation (individual per lamp per year) from the 15-year monitoring data, stratified by year and farm type.

Figure 2—source data 2. Rice brown planthopper: mean and standard deviation (individual per lamp per year) from the 15-year monitoring data, stratified by year and farm type.

Figure 2—source data 3. Rice leaf roller: mean and standard deviation (individual per ha per year) from the 15-year monitoring data, stratified by year and farm type.

Figure 2—source data 4. Economic Injury Levels of pink rice borers, rice brown planthoppers and rice leaf rollers issued by Shanghai Agricultural Technology Extension and Service Center (SATESC). “/” denotes that there is no Economic Injury Level for rice leaf roller in grain-filling stage of rice.

Figure 3—source data 1. Predator abundances: mean and standard deviation (individual per 100 rice clusters per year) from the 15-year monitoring data, stratified by year and farm type.

Figure 5—source data 1. The amount of commercial insecticide and active ingredient sprayed per crop: mean and standard deviation (kg•ha-1) from the 15-year monitoring data, stratified by year and farm type.

Figure 5—source data 2. The number of insecticide spray per crop: mean and standard deviation from the 15-year monitoring data, stratified by year and farm type.

Figure 5—source data 3. Cost–benefit analysis of plant-diversified farms compared with mono-rice farms. All data are mean values from 2001 to 2015; average grain price is 2.163 RMB per kilogram, and average soybean price is 3.873 RMB per kilogram; about 3% of one-hectare rice field was taken by soybeans in the field ridge, and soybean yield is about 25.0 kilograms per hectare rice field. Means±SE.

Figure 5—source data 4. Grain yield: mean and standard deviation (kg•ha-1) from the 15-year monitoring data, stratified by year and farm type.

Figure 6—source data 1. Stem borer: mean and standard deviation (individual per 100 rice clusters) from the common-location-experiments, stratified by year, farm identity, and farm type.

Figure 6—source data 2. Rice plant-hopper: mean and standard deviation (individual per 100 rice clusters) from the common-location-experiments, stratified by year, farm identity, and farm type.

Figure 6—source data 3. Leaf roller: mean and standard deviation (individual per 100 rice clusters) from the common-location-experiments, stratified by year, farm identity, and farm type.

Figure 6—source data 4. Predator: mean and standard deviation (individual per 100 rice clusters) from the common-location-experiments, stratified by year, farm identity, and farm type.

Figure 6—source data 5. Insecticide amount: mean and standard deviation (kg•ha-1) from the common-location-experiments, stratified by year, farm identity, and farm type.

Figure 6—source data 6. Yield: mean and standard deviation (kg•ha-1) from the common-location-experiments, stratified by year, farm identity, and farm type.

Figure 6—figure supplement 1. Predator abundance in the border crop of soybeans and in the neighboring crop of maize sampled in rice field plots. Vertical bars denote SE.

Figure 7—figure supplement 1. Power analysis results for rice plant-hopper occurrence, where the power are estimated for additional experimental sites (left), additional replicates for each farm type–site–year block (middle), and additional years (right), based on the effect size estimated by the mixed-effect model fitted to the common-location experiment data. The horizontal line marks the standard power level of 80%. Vertical line denotes the 95% confidence interval for the power estimates.

Figure 8—figure supplement 1. Power analysis results for rice leaf roller occurrence. See caption of Figure 7—figure supplement 1 for details.

Figure 9—figure supplement 1. Power analysis results for predator abundance. See caption of Figure 7—figure supplement 1 for details.

Figure 10—figure supplement 1. Power analysis results for grain yield. See caption of Figure 7—figure supplement 1 for details.

Figure 11—figure supplement 1. A path diagram depicting the hypothesis on the relationships among the variables considered in this study. “+” indicates increased level and “-“ indicates decreased level.

Figure 12—figure supplement 1. The layout of each rice plots in each community farm and "Z"-style grain yield sampling. On each farm, the rice growing area was divided into 9 paddy plots, which were 60-70m × 25-35m on each plot. Three to six rice field plots of 0.120-0.167 hectares were selected in each community farm to measure the grain yield each year. The rice plants at harvest stage ("Z"-style sampling with 10, one square meter subplots in each plot) were mowed and threshed, and the grain yield per unit area was obtained. “×” and black solid dots denoted rice, and 1-m2 sampling areas for rice grain yield, respectively. The interval between two adjacent black solid dots was about 5 meters.

Figure 13—figure supplement 1. The layout of plant-diversified fields in which a border crop (soybean) was interplanted around the rice fields and a neighboring crop (maize) was interplanted around the soybeans. (A) Drawing plot diagram for plant-diversified fields; and (B) the photograph for plant-diversified fields. “×”, “o” and “§” denoted rice, soybean and maize, respectively. The layout of each control rice field was similar, but without soybeans or maize.
